# Clinical Significance and Integrative Analysis of the SMC Family in Hepatocellular Carcinoma

**DOI:** 10.3389/fmed.2021.727965

**Published:** 2021-08-30

**Authors:** Hui Nie, Yutong Wang, Xuejie Yang, Zhiming Liao, Xiaoyun He, Jianhua Zhou, Chunlin Ou

**Affiliations:** ^1^Department of Pathology, Xiangya Hospital, Central South University, Changsha, China; ^2^Departments of Ultrasound Imaging, Xiangya Hospital, Central South University, Changsha, China; ^3^National Clinical Research Center for Geriatric Disorders, Xiangya Hospital, Central South University, Changsha, China; ^4^Xiangya Lung Cancer Center, Xiangya Hospital, Central South University, Changsha, China

**Keywords:** hepatocellular carcinoma, SMC family, methylation, immune infiltration, biomarker, therapeutic target

## Abstract

Worldwide, hepatocellular carcinoma (HCC) is one of the most malignant cancers with poor prognosis. The structural maintenance of chromosomes (SMC) gene family has been shown to play important roles in human cancers. Nevertheless, the role of SMC members in HCC is not well-understood. In this study, we comprehensively explored the role of the SMC family in HCC using a series of bioinformatic analysis tools. Studies have demonstrated that the mRNA expression levels of *SMC1A, SMC1B, SMC2, SMC4*, and *SMC6* are significantly overexpressed in HCC, and the protein levels of SMC1A, SMC2, SMC3, SMC4, SMC5, and SMC6 are similarly elevated. Moreover, HCC patients with high SMC2 and SMC4 expression levels exhibit poor survival. Using KEGG and GO analyses, we analyzed the enrichment of gene expression in the biological functions and pathways of the SMC family in HCC. Immune infiltration analysis revealed that the expression of the SMC family is closely associated with B cells, CD4+ T cells, CD8+ T cells, macrophages, neutrophils, and DCs. In conclusion, our findings will enhance a more thorough understanding of the SMC family in HCC progression and provide new directions for the diagnosis and treatment of HCC in the future.

## Introduction

Hepatocellular carcinoma (HCC), a primary cancer of the liver, is the most frequent malignant tumor globally (1). The National Center for Health Statistics shows that HCC is the fifth-leading cause of cancer-related deaths worldwide (2, 3). As one of the most aggressive malignant tumors, the effect of surgical treatment is limited and only applicable to a small number of patients with early HCC (4–6). Advanced treatments such as trans-arterial chemoembolization (TACE) have little effect on improving the survival time of patients with HCC (7). Hence, the search for new treatments and prognostic biomarkers to improve survival of patients with HCC is urgently required.

Most DNA-based processes are affected by the structural maintenance of chromosomes (SMC) protein family. As the central regulator of chromosome dynamics, the SMC family can control the cohesion of sister chromatids, chromosomal condensation, DNA replication, DNA repair and transcription (8). There are six members of the SMC family: SMC1-SMC6, and which there are two variants of SMC1, namely SMC1A and SMC1B (9). Six family members form the core of three different multi-subunit protein complexes, of which SMC1 and SMC3 form a v-type heterodimer, which is the main component of the cohesive complex (10). SMC2 and SMC4 are part of a five-subunit lectin complex (11). SMC5 and SMC6 form a complex closed by kleisin, similar to the structure of cohesin and condensin. They often aggregate at sites of DNA double-strand breaks to promote homologous recombination repair, and therefore play an important role in DNA damage responses (12).

Different types of SMC family mutations can change the cohesion or adhesion of DNA, and at the same time affect various life processes involving chromosomal DNA, leading to the occurrence of various cancers. Recent studies have shown that members of the SMC family are widely involved in the pathological progression of tumors. SMC1A is highly expressed in a variety of malignancies, including colorectal and prostate cancer, and acute myelogenous leukemia (AML). It promotes cancer cell invasion and metastasis by influencing epithelial–mesenchymal transition (EMT), releasing inflammatory mediators, and recruiting downstream target molecules, thereby promoting early tumor formation and tumorigenesis (13–16). The meiosis-specific cohesive member SMC1B is necessary for sister chromatid pairing and preventing telomere shortening (8). *SMC1B* is one of the genes related to HPV+ cervical intraepithelial neoplasia (17). In addition, SMC1B is involved in the development of colorectal cancer (18). The deletion of *SMC2*, which is highly expressed in bladder cancer tissues, blocks the G2/M phase of bladder cancer cells, thereby inhibiting tumor growth (19). SMC3 is involved in drug resistance in lung cancer (20) and has an important impact on the prognosis of AML patients (21). SMC4 plays a regulatory role in glioma, breast cancer, lung adenocarcinoma, and other malignancies, and directly or indirectly affects the survival time of patients with tumors (22–24). SMC5 is associated with ovarian cancer progression (25). Compared with normal tissues, SMC6 has also been confirmed to have abnormal expression in lung cancer, sarcoma, and other tumor types (26).

Previous analyses of the SMC family in HCC remain relatively limited. The clinical significance, biological functions, and immune roles of SMC family members in HCC have not yet been reported. Thus, we comprehensively analyzed the expression of each SMC family members in HCC, and their relationship with prognosis, immune infiltration, genetic alteration, and possible mechanisms involving the SMC family in HCC. Using a series of bioinformatic analysis tools, such as Tumor Immune Estimation Resource (TIMER) (27), Human Protein Atlas project (HPA) (28), and Gene expression profiling analysis (GEPIA) (29), we identified the expression of SMC family members in HCC, and Kaplan-Meier plotter was used to evaluate the prognostic value of SMC family members in HCC. In addition, cBioPortal was used to analyze the mutation of SMC family members in HCC, and to download related co-expressed genes for subsequent enrichment. Finally, TIMER was used to analyze relationships between the expression of SMC family members and immune cell infiltration in HCC. Taken together, our findings provide new insights into the prognostic value and biological roles of the SMC family in HCC.

## Methods

### Gene Expression Analysis

Multiple bioinformatics database resources were accessed to explore the expression levels of SMC family members in HCC tissues.

The website tool TIMER is a comprehensive resource based on the TCGA database, which contains 371 HCC samples and 50 normal liver samples. It provides multiple functions including gene expression comparisons with tumor/normal tissues in different cancers, correlation analysis between genes and immune infiltrating cells, survival analysis, and other functionalities (27). The mRNA expression of SMC family members in different cancers or specific cancer subtypes from TIMER, and the log2 [TPM (Transcripts per million)] was applied for log-scale. We also used the TIMER database to analyse the correlation of SMC family members expression with the immune infiltrating cells.

HPA is a large-scale protein research project (28). This allows researchers to map the location of proteins encoded by the SMC family in human tissues and cells. In this study, we analyzed the protein expression of SMC family members in normal and HCC tissues by immunohistochemistry.

GEPIA is a web server that analyzes RNA sequencing data from the GTEx and TCGA database, and allows users to analyze the human cancer gene expression and interaction analyses (29). It provides researchers with interactive customization functions, such as differential expression analysis, pathological stage analysis, and other diversified functions. We used GEPIA database to evaluate the correlations of SMC family expression with the pathological stage of HCC patients.

### Survival Analysis

The Kaplan–Meier database (https://kmplot.com) was used to analyze the relationship between the HCC patients' prognosis and mRNA expression levels of SMC family members. The 370 HCC samples were split into high and low expression group by the mRNA expression value of auto-selected best cutoff to analyze the correlation of SMC family members expression and the overall survival (OS), relapse free survival (RFS), disease-specific survival (DSS), and progression Free Survival (PFS) in HCC. Kaplan-Meier analysis was assessed by log-rank tests, and statistical significance if the *P*-value < 0.05.

### Immune Infiltrating Analysis

TIMER was used to evaluate associations between mRNA expression of SMC family members and HCC immune infiltrating cells that contained CD4+ T cells, CD8+ T cells, B cells, macrophages, neutrophils, and DCs. Correlation R value was calculated by Spearman's algorithm and adjusted by tumor purity. *P*-value < 0.05 was considered to be statistically significant.

### Methylation and Mutation Analysis

The relationship between SMC family expression and DNA methylation values was analyzed using UALCAN (http://ualcan.path.uab.edu), which is a database comprising systematic integration of DNA methylation data and mRNA expression levels in human cancers (30). We predicted DNA methylation changes of SMC family members in 377 HCC samples and 50 normal liver samples using the UALCAN database.

### Co-expression Network Analysis

cBioPortal (http://cbioportal.org), an open comprehensive platform, can be used to analyze multidimensional cancer genomics and clinical data. The threshold of |log2FC| was 1.2, and the *P*-value cutoff was 0.01. Detailed visual analysis was performed using Cytoscape software v.3.7.2. The co-expression gene lists of SMC family were identified by cBioPortal.

The STRING database was used to assess correlations involving SMC genes. Metascape is a powerful tool for investigating the functional annotation of a certain gene list (31). This database enabled us to conduct Gene Ontology (GO) and Kyoto Encyclopedia of Genes and Genomes (KEGG) pathway enrichment analysis for genes co-expressed with SMC members.

## Results

### Aberrant Expression of the SMC Family Members in HCC

**Figure 1:**
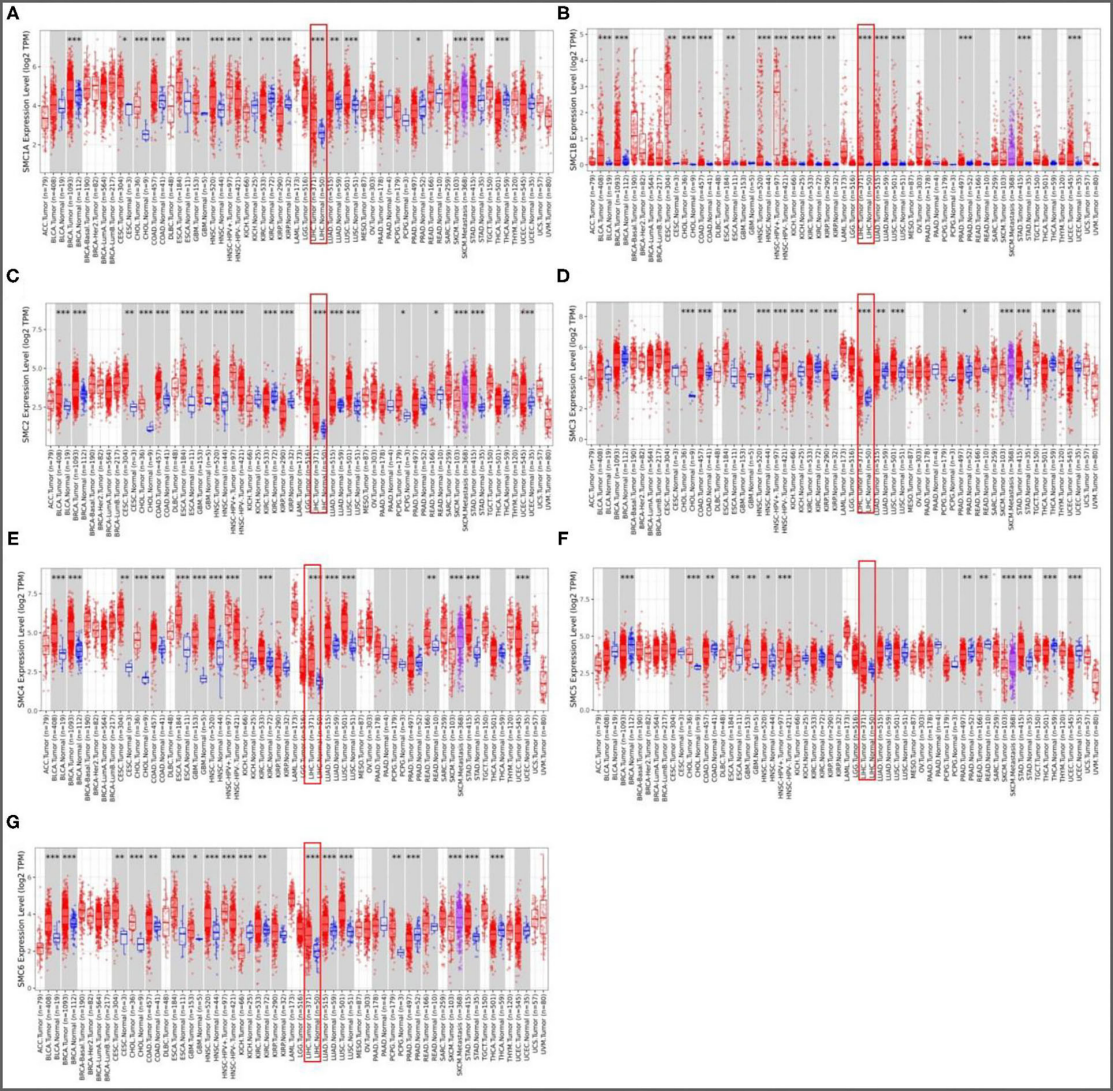
Expression levels of SMC family members in HCC. **(A-G)** Relative expression of *SMC1A, SMC1B, SMC2, SMC3, SMC4, SMC5*, and *SMC6* in different cancers compared with normal tissues by the TIMER database. *P < 0.05, **P < 0.01, ***P < 0.001 compared with control.

**Figure 2:**
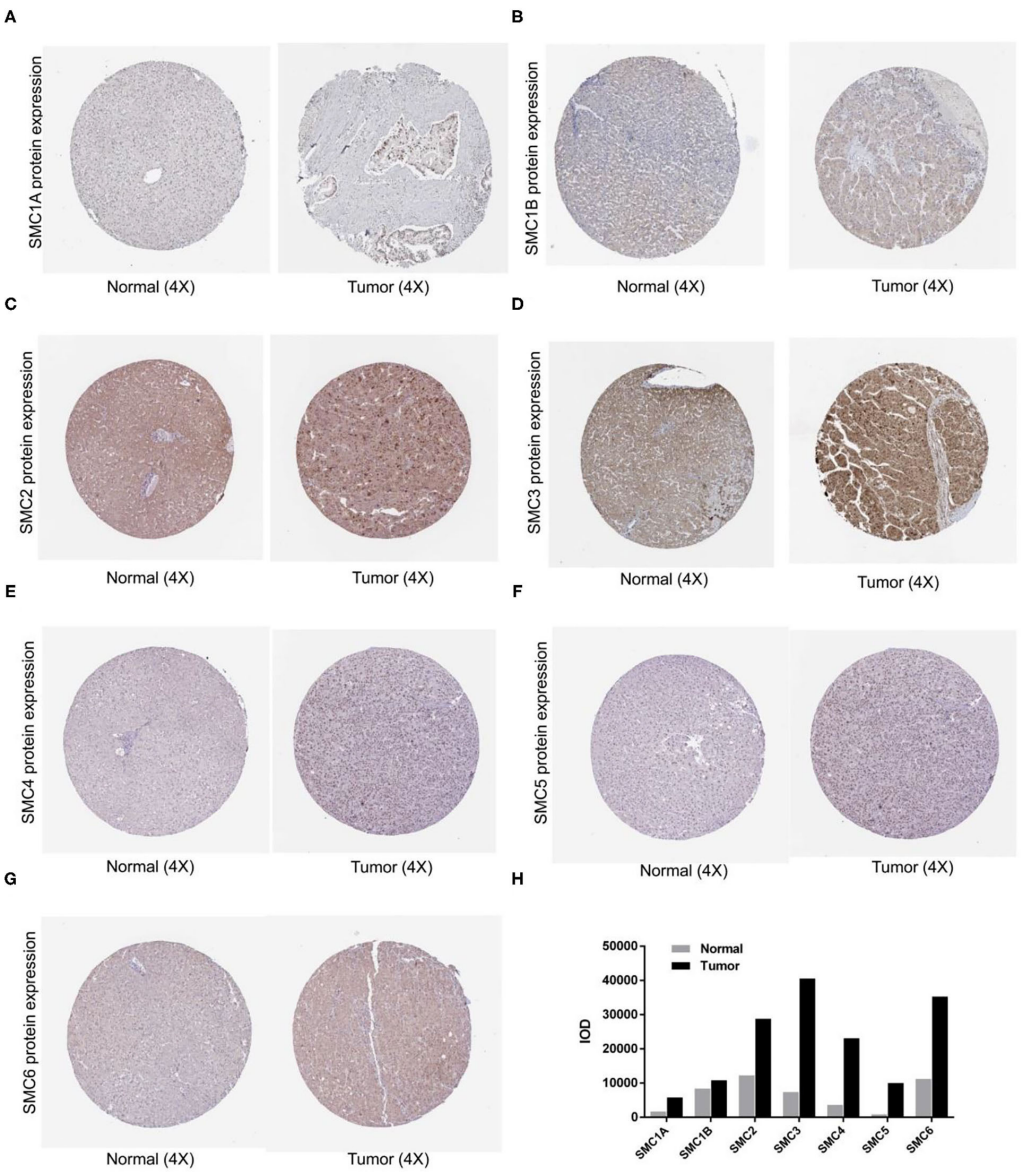
Protein expression levels of SMC family members in HCC. **(A-G)** The HPA database shows the protein expression levels of *SMC1A, SMC1B, SMC2, SMC3, SMC4, SMC5*, and *SMC6* in HCC tissues compared with non-cancerous tissues. **(H)** The integrated optical density (IOD) was used to analyze the expression difference between HCC tissues and noncancerous tissues.

To investigate SMC expression in various tumors compared to normal tissues, transcriptional levels were analyzed using the TIMER database. The levels of *SMC1A, SMC1B, SMC2, SMC3, SMC4*, and *SMC6* mRNA expression were highly upregulated in HCC tissues compared to normal liver tissues, however, *SMC5* showed no statistical difference (Figure 1). To verify these results, we further explored the immunohistochemical staining results of SMC family members from the HPA database. As shown in Figure 2, we found that SMC1A, SMC2, SMC3, SMC4, SMC5, and SMC6 are highly expressed in HCC but not so in normal samples; however, compared with their non-elevated expression in normal liver tissues, SMC1B were weakly expressed in HCC.

### Correlation of the Expression of SMC Family Members With Clinicopathologic Features of HCC Patients

**Figure 3:**
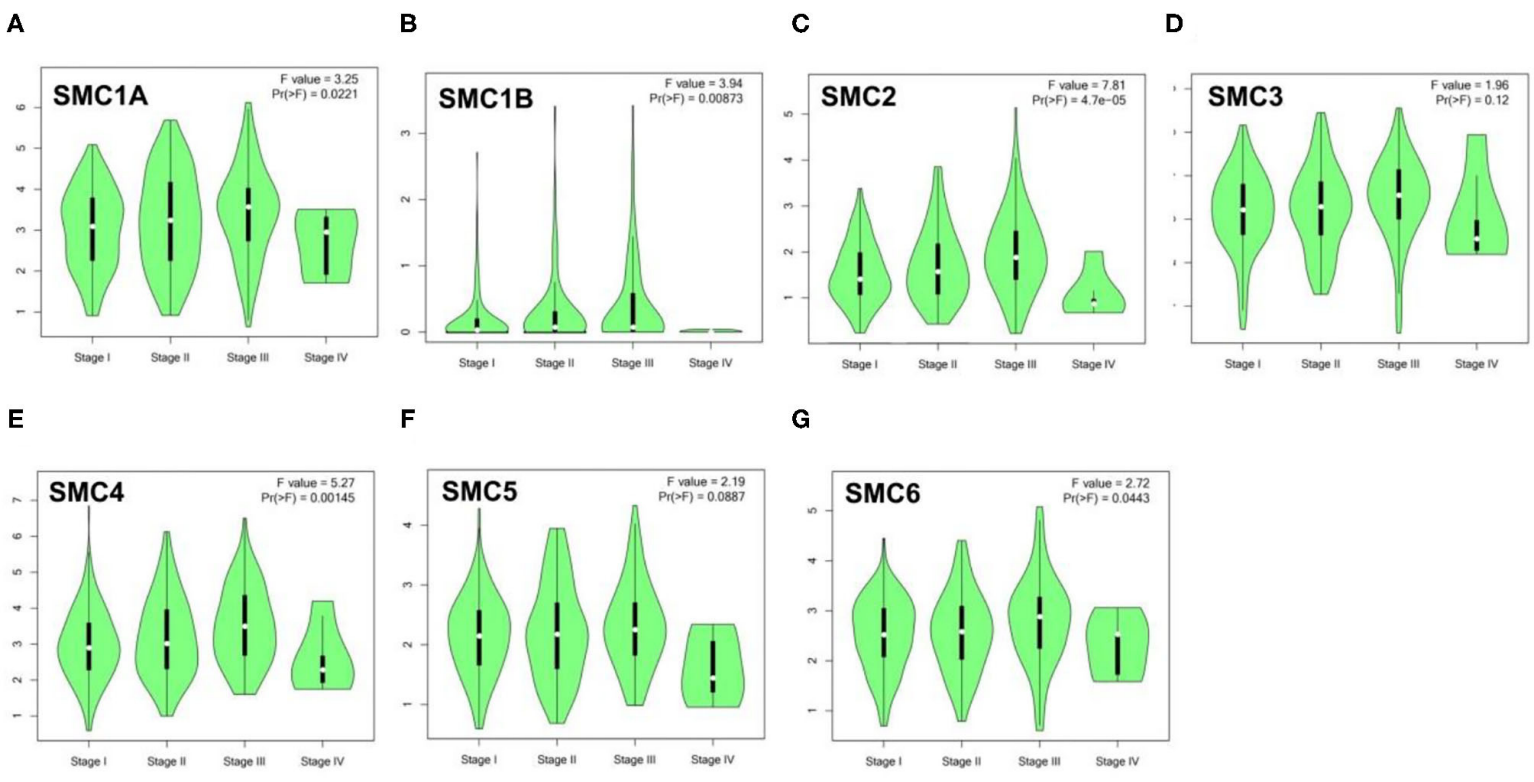
Relationship between the expression level of SMC family members and HCC tumor staging. **(A-G)** GEPIA database was used to evaluate the correlations of the expression of *SMC1A, SMC1B, SMC2, SMC3, SMC4, SMC5*, and *SMC6* with the pathological stage of HCC patients.

Next, we sought to determine whether the expression levels of SMC family members correlate with the patient tumor staging. Correlation analysis of TCGA data using the GEPIA database revealed that the pathological stages of HCC patients are correlated with *SMC1A, SMC1B, SMC2, SMC4*, and *SMC6* expression (Figure 3).

Moreover, we used the TCGA HCC patient samples to further analyze the clinicopathologic parameters and the expression of SMC family members. We found that SMC family members expression are closely associated with the clinicopathologic parameters of HCC patients (Table 1). For example, the high levels of *SMC1B, SMC2*, and *SMC3* expression were significantly associated with the T stage of HCC patients, the high levels of *SMC2, SMC3*, and *SMC4* expression were significantly associated with the N stage of HCC patients, and the high levels of *SMC2* expression was significantly associated with the M stage of HCC patients. These results indicated that SMC family members may serve as potential diagnostic markers for HCC.

**Table 1:** Clinicopathologic parameters and the expression of SMC family members in HCC.

**Characteristics**	***N***	**SMC1A**			**SMC1B**			**SMC2**			**SMC3**			**SMC4**			**SMC5**			**SMC6**		
		**Low**	**High**	***P***	**Low**	**High**	***P***	**Low**	**High**	***P***	**Low**	**High**	***P***	**Low**	**High**	***P***	**Low**	**High**	***P***	**Low**	**High**	***P***
**Gender**				**0.000**			0.998			0.358			**0.040**			0.084			0.406			0.230
Male	233	161	72		186	47		153	80		147	86		155	78		133	100		140	93	
Female	109	47	62		87	22		66	43		56	53		62	47		57	52		58	51	
**Age (year)**				0.143			**0.019**			0.122			0.128			**0.046**			0.105			**0.003**
≤ 60	170	110	60		127	43		102	68		94	76		99	71		87	83		85	85	
>60	172	98	74		146	26		117	55		109	63		118	54		103	69		113	59	
**T stage**				0.176			**0.039**			**0.008**			**0.022**			0.150			0.759			0.087
T1 + T2	256	161	95		211	45		175	82		161	95		168	88		141	115		155	101	
T3 + T4	86	47	39		62	24		45	41		42	44		49	37		49	37		43	43	
**N stage**				0.248			0.181			**0.007**			**0.021**			**0.004**			0.582			0.598
Nx	87	59	28		67	20		67	20		61	26		66	21		45	42		54	33	
N0	251	147	104		204	47		150	101		141	110		150	101		142	109		142	109	
N1	4	2	2		2	2		2	2		1	3		1	3		3	1		2	2	
**M stage**				0.688			0.895			**0.048**			0.579			0.621			0.161			0.148
Mx	77	49	28		61	16		58	19		49	28		52	25		36	41		50	27	
M0	261	156	105		208	53		159	103		151	110		162	99		151	110		147	114	
M1	4	3	1		4	0		3	1		3	1		3	1		3	1		1	3	
**Pathologic stage**				0.149			**0.036**			**0.007**			**0.009**			0.070			0.621			0.077
Stage I + II	252	159	93		208	44		172	80		160	92		167	85		138	114		153	99	
Stage III + IV	90	49	41		65	25		47	43		43	47		50	40		52	38		45	45	
**Histologic grade**				0.212			0.053			**0.002**			**0.009**			**0.003**			0.085			0.230
grade 1 + 2	213	135	78		177	36		150	63		138	75		148	65		126	87		118	95	
grade 3 + 4	129	73	56		96	33		69	60		65	64		69	60		64	65		80	49	

*Bold font indicates significant difference*.

### The Prognostic Value of the SMC Family in HCC Patients

**Figure 4:**
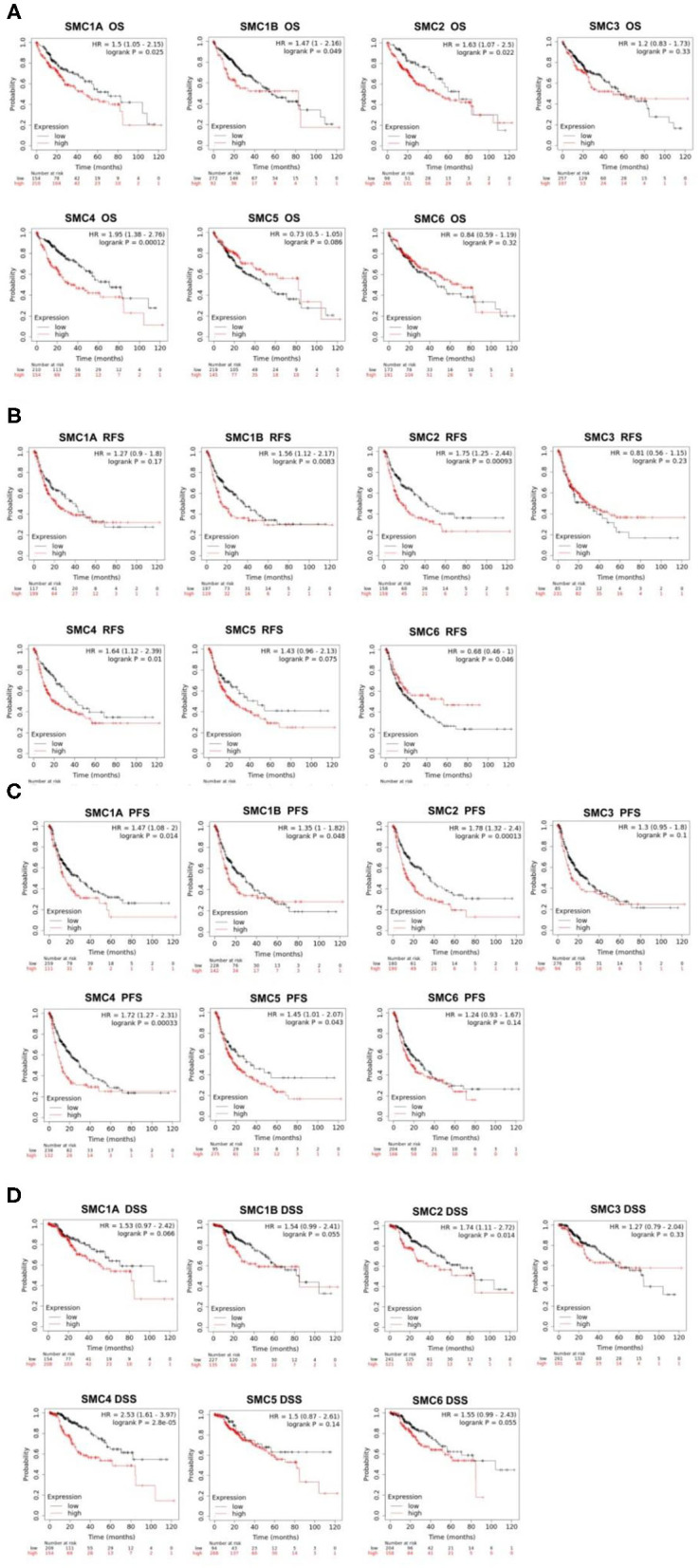
Prognostic value of SMC family in HCC. **(A-D)** The expression of SMC family members showed significant associations with OS, RFS, PFS, and DSS in HCC, and statistical significance was assessed by log-rank tests.

Then, we used the Kaplan-Meier plotter tool was used to perform survival analysis in 364 TCGA HCC patient samples with complete survival and SMC family members expression data. OS was applied for the output of clinical prognosis, and we found that high levels of *SMC1A, SMC1B, SMC2*, and *SMC4* expression were significantly associated with poorer OS in HCC patients (Figure 4A). Furthermore, we discovered that increased *SMC1B, SMC2*, and *SMC4* expression was significantly associated with poorer RFS in HCC (Figure 4B). *SMC1A, SMC1B, SMC2, SMC4*, and *SMC5* abundance were strongly associated with PFS (Figure 4C). Moreover, higher expression of *SMC2* and *SMC4* were significantly associated with poor DSS (Figure 4D). Collectively, SMC family members expression are closely associated with the prognosis of HCC patients. Among them, the relationship between SMC2 and SMC4 expression and clinical prognosis of HCC is closer than others. In our results, the high expressions of *SMC2* and *SMC4* were significantly associated with the poor OS/PFS/RFS/DSS in HCC patients, which indicated that SMC2 and SMC4 may serve as potential prognostic markers for HCC.

We further analyzed the prognostic value of SMC family members in different pathological grades and clinical stages of HCC using the Kaplan–Meier plotter (Table 2). Taking *SMC4* as an example, the mRNA expression of *SMC4* was closely related to poorer OS in grade I, grade II, stage I and stage II, AJCC-T1, and AJCC-T2 HCC patients. Moreover, *SMC1A* and *SMC2* transcriptional expression was significantly associated with worse OS in stage I and stage II HCC patients. We also found that high expression of *SMC1B* was correlated with poor OS in grade I grade II and AJCC-T2 HCC patients. *SMC2* abundance was significantly associated with shorter OS in stage I and stage II and AJCC-T2 HCC patients, and *SMC6* expression was significantly correlated with poorer OS in AJCC-T1 HCC patients. Taken together, these results indicated that a part of SMC family member may serve as a potential prognosis factor in HCC, especially valuable for the OS prediction in HCC patients diagnosed with early stage or low grade.

**Table 2:** Kaplan–Meier plotter was used to analyze the prognostic value of SMC family members in different pathological grades and clinical stages of HCC.

	**SMC1A**		**SMC1B**		**SMC2**		**SMC3**		**SMC4**		**SMC5**		**SMC6**	
	**HR**	***P***	**HR**	***P***	**HR**	***P***	**HR**	***P***	**HR**	***P***	**HR**	***P***	**HR**	***P***
Stage 1 + 2	1.81(1.07-3.06)	**0.025**	1.57(0.92-2.69)	0.096	1.96(1.09-3.53)	**0.023**	1.41(0.87-2.29)	0.164	1.9(1.18-3.08)	**0.008**	1.78(0.95-3.36)	0.069	0.71(0.44-1.15)	0.016
Stage 3 + 4	0.6(0.34-1.07)	0.08	0.63(0.35-1.15)	0.129	1.38(0.76-2.5)	0.281	0.62(0.35-1.11)	0.103	1.63(0.9-2.96)	0.103	0.53(0.28-0.98)	0.039	0.79(0.41-1.54)	0.492
Grade 1	1.44(0.56-3.7)	0.444	0.37(0.14-1.01)	**0.043**	1.64(0.64-4.24)	0.302	3.63(0.81-16.25)	0.074	3.7(1.31-10.49)	**0.009**	0.35(0.11-1.06)	0.053	2.28(0.9-5.77)	0.074
Grade 2	1.66(0.99-2.8)	0.054	1.85(1.08-3.17)	**0.022**	1.63(0.96-2.78)	0.07	1.31(0.78-2.2)	0.313	2.17(1.29-3.66)	**0.003**	1.67(0.9-3.1)	0.102	0.73(0.43-1.25)	0.257
Grade 3	1.72(0.91-3.27)	0.091	0.7(0.38-1.27)	0.238	1.82(1-3.33)	**0.048**	0.81(0.44-1.48)	0.491	1.71(0.94-3.13)	0.077	0.68(0.37-1.24)	0.201	0.47(0.22-1.02)	0.051
AJCC-T1	1.76(0.91-3.41)	0.092	0.65(0.37-1.17)	0.149	0.59(0.31-1.12)	0.1	0.82(0.43-1.57)	0.552	1.79(1-3.22)	**0.047**	0.58(0.3-1.1)	0.089	0.53(0.29-0.94)	**0.029**
AJCC-T2	2.23(0.95-5.23)	0.059	2.16(0.99-4.7)	**0.047**	4.51(1.36-14.94)	**0.007**	2.09(0.94-4.46)	0.064	2.68(1.29-5.55)	**0.006**	2.87(0.99-8.29)	**0.042**	0.71(0.31-1.6)	0.405
AJCC-T3	0.64(0.35-1.18)	0.15	0.66(0.35-1.24)	0.194	1.39(0.74-2.62)	0.298	0.68(0.37-1.25)	0.215	1.82(0.97-3.39)	0.058	0.55(0.29-1.05)	0.066	1.34(0.68-2.65)	0.394

*Bold font indicates significant difference*.

### Genetic Alterations and Functional Enrichment Analysis of SMC Family Members

**Figure 5:**
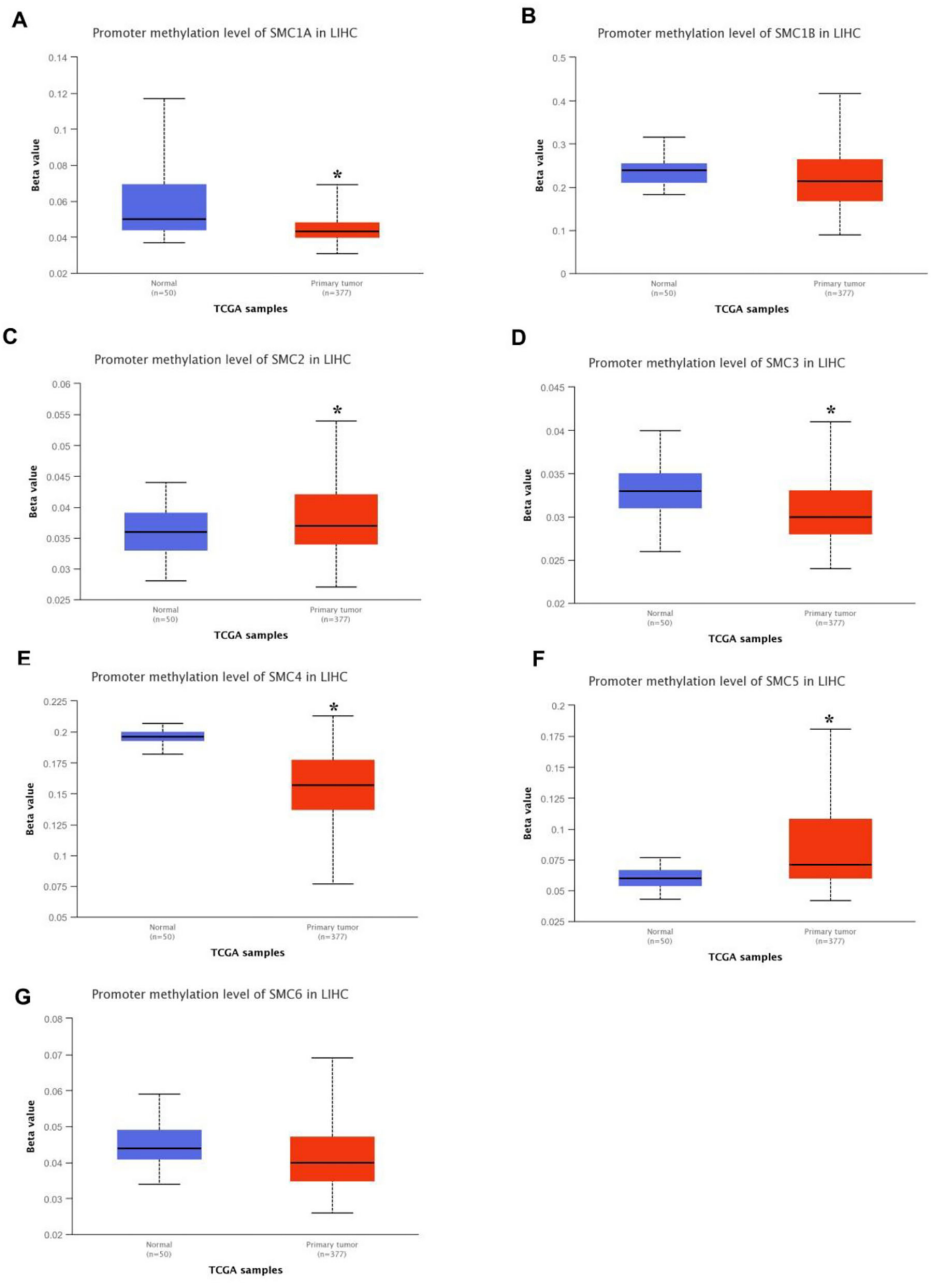
DNA methylation levels of SMC family genes in HCC. **(A-G)** The DNA methylation change of *SMC1A, SMC1B, SMC2, SMC3, SMC4, SMC5*, and *SMC6* in HCC by the UALCAN database. *P < 0.05 compared with control.

It is widely known that genetic alteration plays a key role in the development of cancer. Using the UALCAN database, we identified SMC gene methylation levels in HCC patients. Compared with healthy humans, the DNA methylation levels of *SMC1A, SMC3*, and *SMC4* were significantly lower in HCC samples, whereas *SMC2* and *SMC5* exhibited significantly higher levels in HCC tissues, and other SMC family member exhibited statistically insignificant differences between normal and cancer tissues (Figure 5). These altered DNA methylation levels of SMC gene family members were closely associated with their differences in expression levels, which may provide novel targets for cancer patients by DNA methylation-targeting drugs.

**Figure 6:**
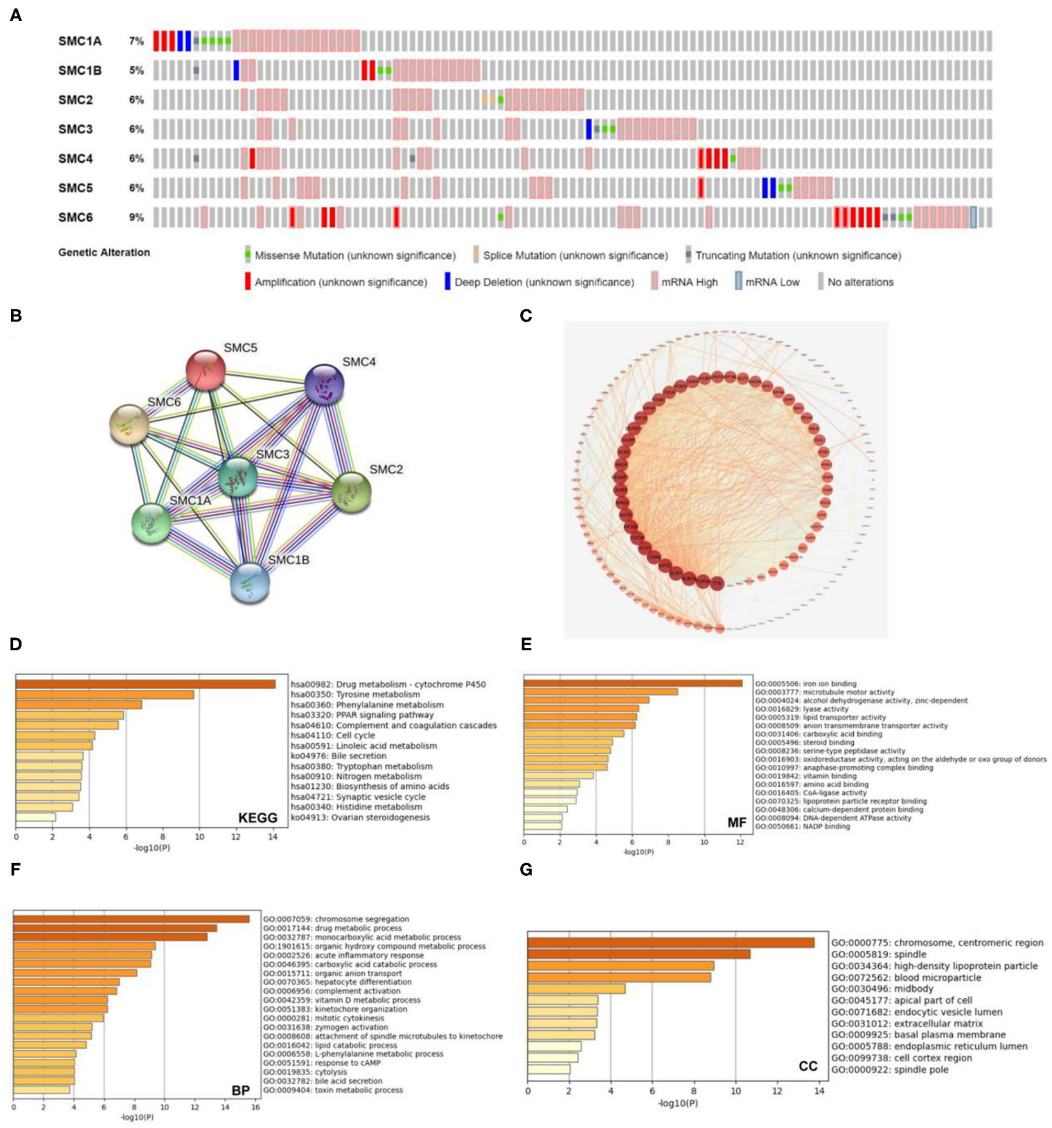
Genetic alterations and pathway enrichment analysis of SMC family in HCC. **(A)** The genetic alteration of *SMC1A, SMC1B, SMC2, SMC3, SMC4, SMC5*, and *SMC6* in HCC was illustrated from top to bottom. **(B)** The SMC family members interaction analysis was evaluated by STRING database. **(C)** Construction a network of genes co-expressed with SMC family members in HCC. **(D-G)** The KEGG enrichment pathway, Molecular functions, biological processes, and cell components of co-expressed genes was analyzed by Metascape database.

Furthermore, we conducted a series of analyses to confirm the genetic alteration status of SMC family using the cBioPortal database. We observed that SMC genes were altered in 103 samples from 360 patients (29%). The highest rate of alteration frequency in *SMC6* was approximately 9%. Among all alterations involving SMC family member genes, the most common alteration types were mRNA alterations (Figure 6A). Moreover, we used the STRING database to construct the protein–protein interaction (PPI) network among the SMC family members, and found that seven SMC family members were all included and served as hub nodes in the interactive network (Figure 6B). We then identified co-expressed genes with a threshold value of |log2FC|> 1.2 and *P* < 0.01 from the cBioportal database (Supplementary Table 1), and presented a map of co-expression networks of key genes associated with the SMC family using Cytoscape_v.3.7.2 (Figure 6C).

In addition, we used the identified co-expressed genes to conduct GO and KEGG pathway analyses on the Metascape database. For KEGG pathway analysis, the drug metabolizing cytochrome p450, tyrosine metabolism, and phenylalanine metabolism were included in these co-expressed genes (Figure 6D). Molecular function analysis indicated that these genes were primarily related to iron ion binding and microtubule motor activity (Figure 6E). Biological process analysis indicated that these genes were mainly involved in chromosome segregation, drug metabolizing processes, and monocarboxylic acid metabolic processes (Figure 6F). Cellular component analysis revealed that these genes were frequently associated with chromosomes, centromeric regions, and spindles (Figure 6G). These results indicated that the function of SMC family might be involved in drug resistance of HCC, thereby affecting the outcome of treatment in HCC patients.

### Association Between the Expression of SMC Family Members and Immune Infiltration in HCC

**Figure 7:**
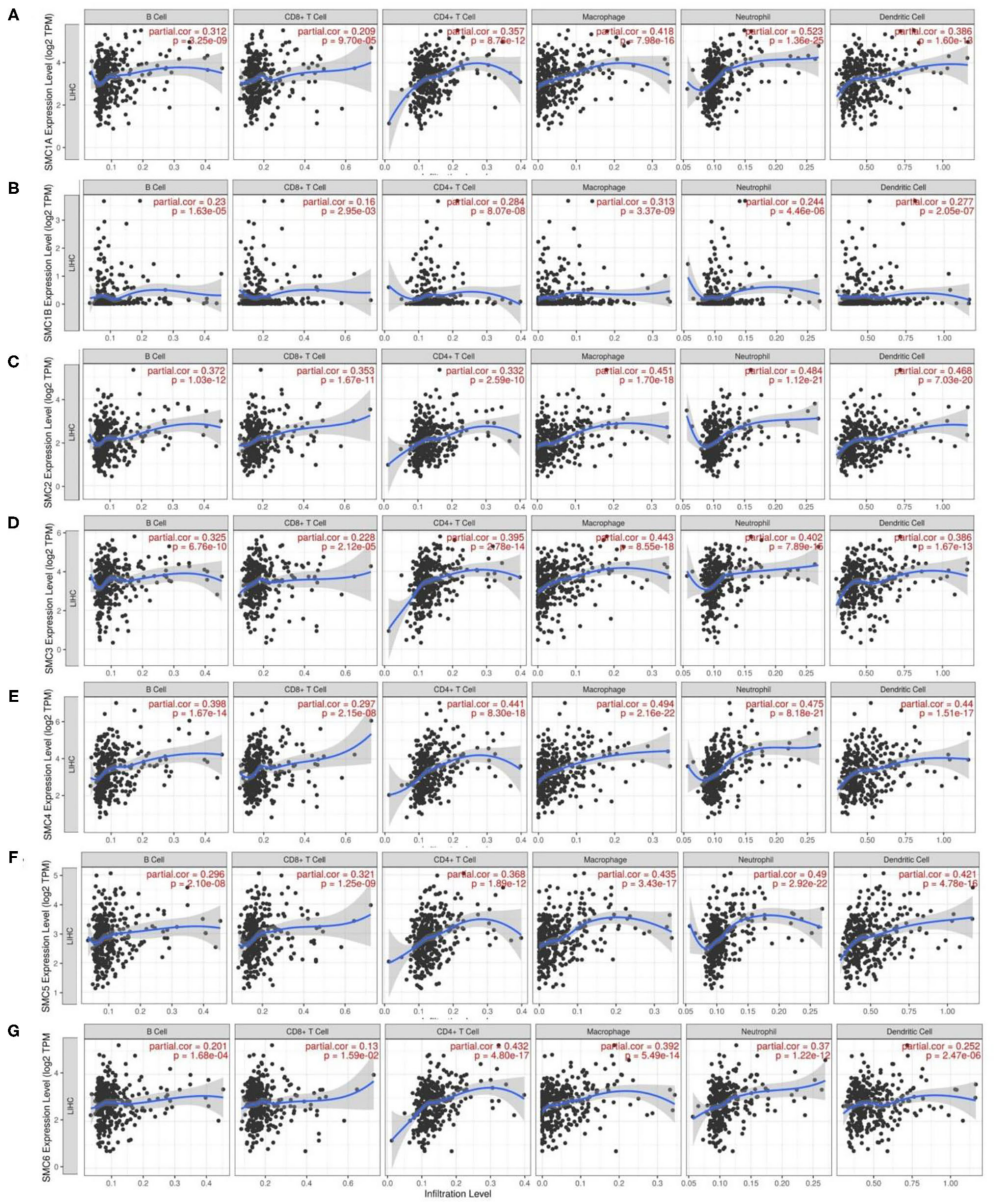
Analysis of correlations involving the expression of SMC family genes and immune cell infiltration in HCC. **(A-G)** Correlation of *SMC1A, SMC1B, SMC2, SMC3, SMC4, SMC5*, and *SMC6* mRNA expression and the immune cell infiltration levels in HCC by the TIMER database.

To further understand the roles of SMC family members in HCC, we utilized the TIMER resource to explore the molecular features of tumor–immune interactions. As shown in Figure 7, transcriptional expression of the SMC family was positively associated with B cells, CD4+ T cells, CD8+ T cells, macrophages, neutrophils, and DCs (r > 0.1, *P* < 0.05). These results suggest the potential effect of SMC family members on the immune response in the tumor microenvironment (TME) of HCC.

Next, we investigated potential correlations between SMC family member expression and immune signature markers of various immune cells infiltrating HCC in the TIMER database (Table 3). We observed that SMC1A levels were strongly associated with tumor-associated macrophages (TAM), M1, M2, neutrophils, Th2, Tregs, and monocytes. SMC1B showed a strong correlation with T cell exhaustion and monocytes. SMC2 expression was highly related to CD8+ T cells, T cells, TAM, M2, neutrophils, DCs, Th1, Th2, Tfh, Tregs, T cell exhaustion, and monocyte infiltration in HCC. SMC3 exhibited better correlation with B cells, TAM, M1, M2, neutrophils, Th1, Th2, Tregs, and monocyte markers. mRNA expression of *SMC4* was only moderately or weakly linked with M1, NKs, Tfh, and Th17 gene markers, but was strongly associated with other immune cells. CD8+ T cells, B cells, T cells, TAM, M2, neutrophils, Th1, Th2, Tregs, monocytes, and T cell exhaustion demonstrated favorable correlations with *SMC5* expression. Moreover, we also observed that the mRNA expression of *SMC6* was strongly correlated with M1, M2, neutrophils, Th2, Tregs, and monocytes. Interestingly, the transcriptional expression of SMC family members was highly associated with monocytes. These results further validated that the SMC family members are relevant to immune-infiltrating cells in HCC.

**Table 3:** The association between the expression of SMC family members and the markers of immune cells.

		**SMC1A**		**SMC1B**		**SMC2**		**SMC3**		**SMC4**		**SMC5**		**SMC6**	
		**Cor**	***P***	**Cor**	***P***	**Cor**	***P***	**Cor**	***P***	**Cor**	***P***	**Cor**	***P***	**Cor**	***P***
CD8+ T cell	CD8A	0.202	^***^	0.135	^*^	0.317	^***^	0.282	^***^	0.311	^***^	0.296	^***^	0.223	^***^
	CD8B	0.104	0.054	0.134	^*^	0.257	^***^	0.159	^**^	0.232	^***^	0.192	^***^	0.092	0.090
	GZMA	0.089	0.100	0.102	0.059	0.191	^***^	0.086	0112	0.195	^***^	0.194	^***^	0.097	0.071
B cell	CD19	0.150	^**^	0.324	^***^	0.269	^***^	0.255	^***^	0.279	^***^	0.207	^***^	0.139	^*^
	CD79A	0.115	^*^	0.186	^**^	0.214	^***^	0.248	^***^	0.261	^***^	0.204	^***^	0.147	^**^
	MS4A1	0.099	0.066	0.125	^*^	0.150	^**^	0.246	^***^	0.237	^***^	0.220	^***^	0.213	^***^
T cell	CD3D	0.093	0.084	0.228	^***^	0.240	^***^	0.140	^**^	0.295	^***^	0.224	^***^	0.119	^*^
	CD3E	0.195	^***^	0.181	^**^	0.273	^***^	0.266	^***^	0.324	^***^	0.293	^***^	0.195	^***^
	CD2	0.152	^**^	0.191	^***^	0.252	^***^	0.236	^***^	0.312	^***^	0.281	^***^	0.188	^***^
TAM	CCL2	0.243	^***^	0.139	^*^	0.262	^***^	0.226	^***^	0.287	^***^	0.297	^***^	0.288	^***^
	CD68	0.315	^***^	0.273	^***^	0.281	^***^	0.347	^***^	0.272	^***^	0.230	^***^	0.155	^**^
	IL10	0.347	^***^	0.234	^***^	0.380	^***^	0.346	^***^	0.390	^***^	0.346	^***^	0.277	^***^
M1	IRF5	0.438	^***^	0.224	^***^	0.352	^***^	0.423	^***^	0.442	^***^	0.356	^***^	0.470	^***^
	PTGS2	0.396	^***^	0.185	^**^	0.375	^***^	0.396	^***^	0.379	^***^	0.428	^***^	0.366	^***^
	NOS2	0.243	^***^	−0.036	0.500	0.137	^*^	0.193	^***^	0.124	^*^	0.141	^**^	0.286	^***^
M2	MS4A4A	0.364	^***^	0.178	^**^	0.324	^***^	0.280	^***^	0.273	^***^	0.312	^***^	0.199	^***^
	CD163	0.425	^***^	0.176	^**^	0.361	^***^	0.341	^***^	0.272	^***^	0.322	^***^	0.285	^***^
	VSIG4	0.344	^***^	0.178	^**^	0.307	^***^	0.235	^***^	0.269	^***^	0.303	^***^	0.216	^***^
Neutrophils	ITGAM	0.447	^***^	0.250	^***^	0.397	^***^	0.277	^***^	0.402	^***^	0.358	^***^	0.273	^***^
	CCR7	0.243	^***^	0.173	^**^	0.251	^***^	0.305	^***^	0.291	^***^	0.314	^***^	0.253	^***^
	SIGLEC5	0.504	^***^	0.247	^***^	0.494	^***^	0.492	^***^	0.471	^***^	0.444	^***^	0.401	^***^
DC	HLA-DQB1	0.171	^**^	0.108	^*^	0.199	^***^	0.151	^**^	0.202	^***^	0.144	^**^	0.092	0.089
	HLA-DPB1	0.265	^***^	0.137	^**^	0.295	^***^	0.257	^***^	0.262	^***^	0.252	^***^	0.129	^*^
	HLA-DRA	0.354	^***^	0.178	^**^	0.350	^***^	0.336	^***^	0.315	^***^	0.304	^***^	0.194	^***^
	HLA-DPA1	0.377	^***^	0.154	0.004	0.340	^***^	0.331	^***^	0.311	^***^	0.302	^***^	0.225	^***^
	ITGAX	0.433	^***^	0.307	^***^	0.404	^***^	0.381	^***^	0.498	^***^	0.363	^***^	0.351	^***^
	CD1C	0.230	^***^	0.070	0.193	0.239	^***^	0.361	^***^	0.282	^***^	0.271	^***^	0.309	^***^
	NRP1	0.576	^***^	0.166	^**^	0.510	^***^	0.652	^***^	0.418	^***^	0.466	^***^	0.451	^***^
NK cell	KIR2DL1	0.122	^*^	0.012	0.825	0.053	0.324	0.021	0.696	0.017	0.752	0.106	^*^	0.030	0.583
	KIR2DL3	0.205	^***^	0.107	^*^	0.272	^***^	0.181	^**^	0.233	^***^	0.220	^***^	0.142	^**^
	KIR2DL4	0.195	^***^	0.167	^**^	0.232	^***^	0.112	^*^	0.206	^***^	0.194	^***^	0.066	0.223
	KIR3DL1	0.209	^***^	0.013	0.808	0.187	^***^	0.162	^**^	0.104	0.054	0.135	^*^	0.154	^**^
	KIR3DL2	0.112	^*^	0.073	0.178	0.184	^**^	0.153	^**^	0.135	^*^	0.157	^**^	0.125	^*^
	KIR3DL3	0.049	0.361	0.059	0.271	0.010	0.858	0.033	0.540	0.044	0.418	0.013	0.815	−0.022	0.685
	KIR2DS4	0.105	0.052	0.069	0.198	0.156	^**^	0.112	^*^	0.111	^*^	0.183	^**^	0.060	0.265
Th1	TBX21	0.164	^**^	0.063	0.241	0.244	^***^	0.225	^***^	0.227	^***^	0.250	^***^	0.187	^***^
	STAT1	0.468	^***^	0.210	^***^	0.471	^***^	0.535	^***^	0.509	^***^	0.450	^***^	0.447	^***^
	STAT4	0.210	^***^	0.244	^***^	0.246	^***^	0.217	^***^	0.381	^***^	0.366	^***^	0.280	^***^
	IFNG	0.143	^**^	0.197	^***^	0.299	^***^	0.200	^***^	0.294	^***^	0.202	^***^	0.181	^**^
Th2	STAT6	0.379	^***^	0.096	0.075	0.295	^***^	0.425	^***^	0.255	^***^	0.381	^***^	0.321	^***^
	GATA3	0.337	^***^	0.240	^***^	0.334	^***^	0.328	^***^	0.383	^***^	0.373	^***^	0.319	^***^
	STAT5A	0.430	^***^	0.267	^***^	0.447	^***^	0.409	^***^	0.386	^***^	0.401	^***^	0.317	^***^
	IL13	0.107	^*^	0.142	^**^	0.109	^*^	0.006	0.906	0.186	^**^	0.148	*8	0.228	^***^
Tfh	BCL6	0.422	^***^	0.134	^*^	0.396	^***^	0.494	^***^	0.334	^***^	0.367	^***^	0.359	^***^
	IL21	0.165	^**^	0.097	0.073	0.198	^***^	0.173	^**^	0.143	^**^	0.141	^**^	0.100	0.062
Th17	STAT3	0.557	^***^	0.189	^***^	0.396	^***^	0.466	^***^	0.355	^***^	0.456	^***^	0.379	^***^
	IL17A	0.098	0.069	0.045	0.402	0.129	^*^	0.182	^**^	0.137	^*^	0.181	^**^	0.115	^*^
Treg	FOXP3	0.385	^***^	0.264	^***^	0.323	^***^	0.312	^***^	0.370	^***^	0.275	^***^	0.413	^***^
	STAT5B	0.664	^***^	0.183	^**^	0.527	^***^	0.720	^***^	0.458	^***^	0.504	^***^	0.642	^***^
	CCR8	0.538	^***^	0.292	^***^	0.504	^***^	0.564	^***^	0.601	^***^	0.478	^***^	0.500	^***^
	TGFB1	0.310	^***^	0.288	^***^	0.353	^***^	0.389	^***^	0.404	^***^	0.321	^***^	0.268	^***^
T-cell exhaustion	PDCD1	0.164	^**^	0.267	^***^	0.279	^***^	0.261	^***^	0.348	^***^	0.254	^***^	0.147	^**^
	CTLA4	0.164	^**^	0.230	^***^	0.308	^***^	0.235	^***^	0.359	^***^	0.274	^***^	0.130	^*^
	HAVCR2	0.403	^***^	0.321	^***^	0.441	^***^	0.344	^***^	0.484	^***^	0.397	^***^	0.268	^***^
	LAG3	0.107	^*^	0.229	^***^	0.259	^***^	0.155	^**^	0.267	^***^	0.193	^***^	0.164	^**^
Monocyte	CD86	0.407	^***^	0.286	^***^	0.451	^***^	0.390	^***^	0.462	^***^	0.408	^***^	0.287	^***^
	C3AR1	0.439	^***^	0.230	^***^	0.421	^***^	0.377	^***^	0.389	^***^	0.393	^***^	0.276	^***^
	CSF1R	0.365	^***^	0.187	^***^	0.363	^***^	0.317	^***^	0.318	^***^	0.337	^***^	0.213	^***^

*Correlation R value was calculated by Spearman's algorithm and adjusted by tumor purity*.

* *P < 0.05*,

** *P <0.01*,

*** *P < 0.001*.

## Discussion

Our research comprehensively explained the biological functions of each member of the SMC family in HCC from five different aspects: mRNA and protein expression levels, disease prognosis, gene mutations, immune infiltration, and pathway analysis.

In the SMC family, SMC1 and SMC3 and the two sister chromatid cohesive (SCC) proteins SCC1 and SCC3 together constitute the core of the cohesin subunit. This complex mainly maintains chromatin equalization during mitosis, DNA damage repair, and other important biological functions. SMC2 and SMC4, the core components of the condensin complex, are required for successful separation during chromosome assembly and cell division. Unlike SMC1/3 or SMC2/4, SMC5/SMC6 forms a third complex that is important for DNA repair and faithful replication. A previous study revealed that certain members of the SMC family are abnormally expressed in HCC tissues. For example, SMC1A was first confirmed to be expressed at an abnormally high level in human liver cancer cell lines (32). Chen et al. demonstrated that SMC2 is highly expressed in HCC (33), and abnormal elevation of SMC4 is observed in HCC tissues (34). To the best of our knowledge, the present study is the first to report the expression of each member of the SMC family in HCC tissues. In addition, consistent with previous research results, we also found that *SMC1A, SMC1B, SMC2, SMC3, SMC4*, and *SMC6* were overexpressed at the mRNA level in HCC when compared with non-tumor cells. Meanwhile, the protein levels of SMC1A, SMC2, SMC3, SMC4, SMC5, and SMC6 were elevated in HCC.

To date, few specific studies have been conducted concerning potential relationships involving SMC family members and HCC clinical significance. Phosphorylation of SMC1A promotes the invasion and metastasis of liver cancer cells. In addition, the SMC1A phosphorylation is negatively correlated with the prognosis of patients with Tumor Node Metastasis (TNM) liver cancer stages III and IV (32). SMC4 acts as a direct target of miR-219 and can inhibit liver cancer cell proliferation, migration, and invasion (35). Next, we further explored the clinical correlation and prognostic value of abnormally expressed SMCs in HCC patients. We found a correlation between the expression levels of *SMC1A, SMC1B, SMC2, SMC4*, and *SMC6* and clinicopathological staging for the first time. Furthermore, the overexpression of *SMC1A* and *SMC1B* was significantly associated with poor OS and PFS, while elevated expression of *SMC2* and *SMC4* was associated with poorer OS, RFS, PFS, and DSS in HCC patients. Our results also showed that increased *SMC5* mRNA expression was associated with poor RFS and PFS, but was further associated with more prolonged OS. The high expression of *SMC6* resulted in significantly better RFS and DSS. Thus, combining additional clinical data to explore prognosis is also a direction for our follow-up research. Overall, members of the SMC family, such as *SMC2* and *SMC4*, have great potential in predicting patient prognosis and as diagnostic markers in patients with HCC.

Here, mutation analysis comprehensively revealed a number of genetic changes involving SMC family members in HCC. These results revealed that all members of the SMC family mainly exhibited mRNA alterations in HCC. KEGG pathway enrichment and GO analyses were used to explore the potential biological functions involved. Pathway enrichment analysis revealed that the drug-metabolizing cytochrome P450 pathway, tyrosine metabolism, PPAR signaling pathway, DNA-dependent ATPase activity, and other drug therapeutic-related signaling pathways were related to SMC family members in HCC. The cytochrome P450 pathway can oxidize many anticancer drugs, causing changes in drug metabolism and thus influencing the process of tumor treatment and drug resistance (36). Studies have shown that P450 plays an important role in the drug therapy of prostate cancer (37). As a classical tumor-related signaling pathway, receptor tyrosine kinase (RTK) is involved in drug metabolism in many cancers, including non-small cell carcinoma, and has become a major anticancer pathway for the purpose of metabolic therapy (38). Peroxisome proliferator-activated receptor (PPAR), which is expressed in many human solid tumors, is a member of the nuclear receptor superfamily and is considered an important therapeutic target (39). Hence, we believe that the SMC family may represent new targets in HCC therapy through the interaction of key molecules with drug-related signaling pathways.

Although the principles of effective liver cancer treatment have been clearly established, the survival time of patients with advanced liver cancer has not been significantly improved. In addition to traditional surgical intervention, chemotherapy, radiotherapy, and other treatment modalities, immunotherapy has become a new means of HCC treatment. Currently, there is only a small amount of literature suggesting that members of the SMC family are associated with immune cells in different types of diseases. For example, SMC3 is involved in the transport of germinal centers related to B cells and plays an important role in limiting its malignant transformation (40). Deletion of *SMC4* inhibits the production of pro-inflammatory cytokines such as TNF-α and IL-6 in macrophages (41). In addition, the SMC5/6 complex has been shown to be involved in a range of immune responses induced by Hepatitis B virus (HBV) infection (42). However, there have been no reports regarding relationships between SMC proteins and immunotherapy. Immune infiltration analysis revealed relationships involving SMC family members and the HCC microenvironment to some extent. Unexpectedly, our results showed that all members of the SMC family are related to six types of immune cells, including B cells, CD4+ T cells, CD8+ T cells, macrophages, neutrophils, and DCs. Therefore, we investigated the association between the expression of SMC family members and markers of immune infiltrates in HCC. Surprisingly, the expression of SMC family members is strongly associated with multiple immune cells, including M2 cells. Among them, M2 cells play a positive role in the occurrence and development of cancer (43). Moreover, CD4+ CD25+ FOXP3 regulatory T cells (TREG) is one of the most studied cell population in the study of tumor microenvironment, which displayed the immunosuppressive role and could be a target of immunotherapy in the HCC (44). However, due to the limitation of TIMER database, we didn't analyze the correlation between the expression of SMC family genes and TREG infiltration in HCC, and will further explore their relationship in the subsequent study. These results indicated that SMC family members may play vital roles in the local tumor microenvironment in HCC and might be used as multiple targets for immunotherapeutic strategies in HCC in the future.

In conclusion, we systematically analyzed the expression and prognostic value of SMC family members in HCC and provided a thorough understanding of immune roles involving the SMC family in HCC. Our results indicated that the SMC family may play vital roles in tumor progression and immune responses in HCC patients, which may be beneficial for the development of superior diagnostic and treatment modalities for HCC patients in order to improve their prognosis.

## Data Availability Statement

The datasets presented in this study can be found in online repositories. The names of the repository/repositories and accession number(s) can be found in the article/Supplementary Material.

## Author Contributions

HN and YW for acquisition of data, analysis and interpretation of data, statistical analysis, and drafting of the manuscript. CO and JZ conceived and designed the experiments. All authors participated in writing the paper and read and approved the final manuscript.

## Conflict of Interest

The authors declare that the research was conducted in the absence of any commercial or financial relationships that could be construed as a potential conflict of interest.

## Publisher's Note

All claims expressed in this article are solely those of the authors and do not necessarily represent those of their affiliated organizations, or those of the publisher, the editors and the reviewers. Any product that may be evaluated in this article, or claim that may be made by its manufacturer, is not guaranteed or endorsed by the publisher.
